# Crystal structure of 5-bromo-1-ethyl­indoline-2,3-dione

**DOI:** 10.1107/S2056989015023002

**Published:** 2015-12-06

**Authors:** Yassine Kharbach, Amal Haoudi, Frédéric Capet, Ahmed Mazzah, Lahcen El Ammari

**Affiliations:** a Laboratoire de Chimie Appliquée, Faculté des Sciences et Techniques, Université Sidi Mohamed Ben Abdallah, Fès, Morocco; b Unité de Catalyse et de Chimie du Solide (UCCS), UMR 8181. Ecole Nationale, Supérieure de Chimie de Lille, Université Lille 1, 59650 Villeneuve, d’Ascq Cedex, France; c USR 3290 Miniaturisation pour l’Analyse, la Synthèse et la Protéomique, 59655 Villeneuve d’Ascq Cedex, Université Lille 1, France; d Laboratoire de Chimie du Solide Appliquée, Faculté des Sciences, Université Mohammed V, Avenue Ibn Battouta, BP 1014, Rabat, Morocco

**Keywords:** crystal structure, indoline, C—H⋯O hydrogen bonds, slipped parallel π–π inter­action, short Br⋯O inter­action

## Abstract

The title compound, C_10_H_8_BrNO_2_, crystallizes with two independent molcules (*A* and *B*) in the asymmetric unit. In each mol­ecule, the indoline ring system is almost planar, with the largest deviation from the mean plane being 0.016 (2) Å in mol­ecule *A* and 0.040 (13) Å in mol­ecule *B*. In each mol­ecule, the ethyl group is nearly perpendicular to the indoline ring system with C—C—N—C torsion angles of −94.8 (3) and 93.0 (3)° in mol­ecules *A* and *B*, respectively. In the crystal, the two mol­ecules are inclined to each other, making a dihedral angle of 6.28 (8)°. In the molecular packing, the *A* and *B* mol­ecules are linked by C—H⋯O hydrogen bonds, forming –*A*–*B*–*A*–*B*– chains along [01-1]. Parallel chains are linked *via* a weak slipped parallel π–π inter­action [inter-centroid distance = 3.6107 (14) Å] and a short Br⋯O contact [3.183 (2) Å], forming a three-dimensional structure.

## Related literature   

For biological activities of isatin derivatives, see: Samus *et al.* (2004 ▸); Sarangapani & Reddy (1994 ▸); Varma *et al.* (2004 ▸); Pandeya *et al.* (1999 ▸). For the use of isatin derivatives as reagents in organic synthesis and as raw materials for drug synthesis, see: Abele *et al.* (2003 ▸). For their use as corrosion inhibitors, see: Da Silva *et al.* (2013 ▸).
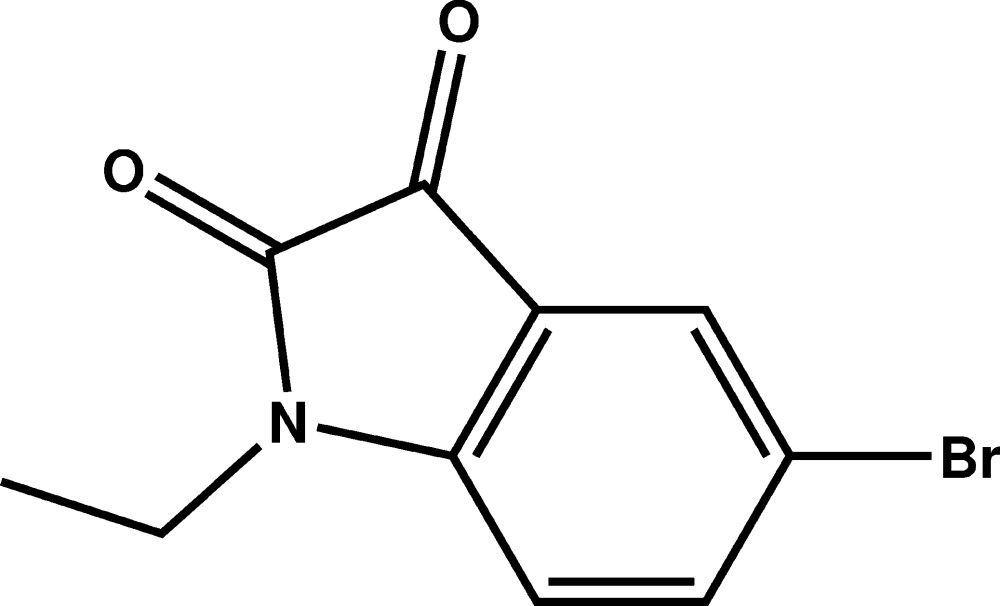


## Experimental   

### Crystal data  

C_10_H_8_BrNO_2_
*M* *_r_* = 254.08
Triclinic, 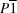

*a* = 9.5198 (3) Å
*b* = 10.0655 (3) Å
*c* = 11.2341 (3) Å
α = 70.9288 (16)°
β = 75.4109 (16)°
γ = 85.2199 (16)°
*V* = 984.58 (5) Å^3^
*Z* = 4
Mo *K*α radiation
μ = 4.15 mm^−1^
*T* = 296 K
0.50 × 0.27 × 0.16 mm

### Data collection  

Bruker APEXII CCD diffractometer
Absorption correction: multi-scan (*SADABS*; Bruker, 2009 ▸) *T*_min_ = 0.363, *T*_max_ = 0.746
40192 measured reflections
6275 independent reflections
4053 reflections with *I* > 2σ(*I*)
*R*_int_ = 0.045

### Refinement  

*R*[*F* ^2^ > 2σ(*F* ^2^)] = 0.039
*wR*(*F* ^2^) = 0.105
*S* = 1.02
6275 reflections
255 parameters
H-atom parameters constrained
Δρ_max_ = 0.66 e Å^−3^
Δρ_min_ = −0.47 e Å^−3^

### 

Data collection: *APEX2* (Bruker, 2009 ▸); cell refinement: *SAINT* (Bruker, 2009 ▸); data reduction: *SAINT*; program(s) used to solve structure: *SHELXS2014* (Sheldrick, 2008 ▸); program(s) used to refine structure: *SHELXL2014* (Sheldrick, 2015 ▸); molecular graphics: *ORTEP-3 for Windows* (Farrugia, 2012 ▸) and *Mercury* (Macrae *et al.*, 2008 ▸); software used to prepare material for publication: *PLATON* (Spek, 2009 ▸) and *publCIF* (Westrip, 2010 ▸).

## Supplementary Material

Crystal structure: contains datablock(s) I. DOI: 10.1107/S2056989015023002/su5250sup1.cif

Structure factors: contains datablock(s) I. DOI: 10.1107/S2056989015023002/su5250Isup2.hkl

Supporting information file. DOI: 10.1107/S2056989015023002/su5250Isup3.cml

**Fig. 1.**
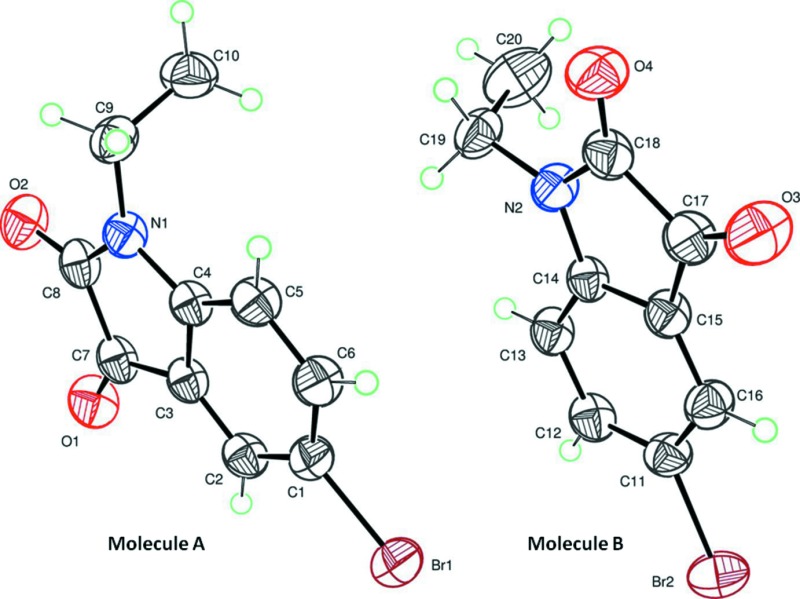
Molecular structure of the title compound, with atom labelling. Displacement ellipsoids are drawn at the 50% probability level.

**Fig. 2.**
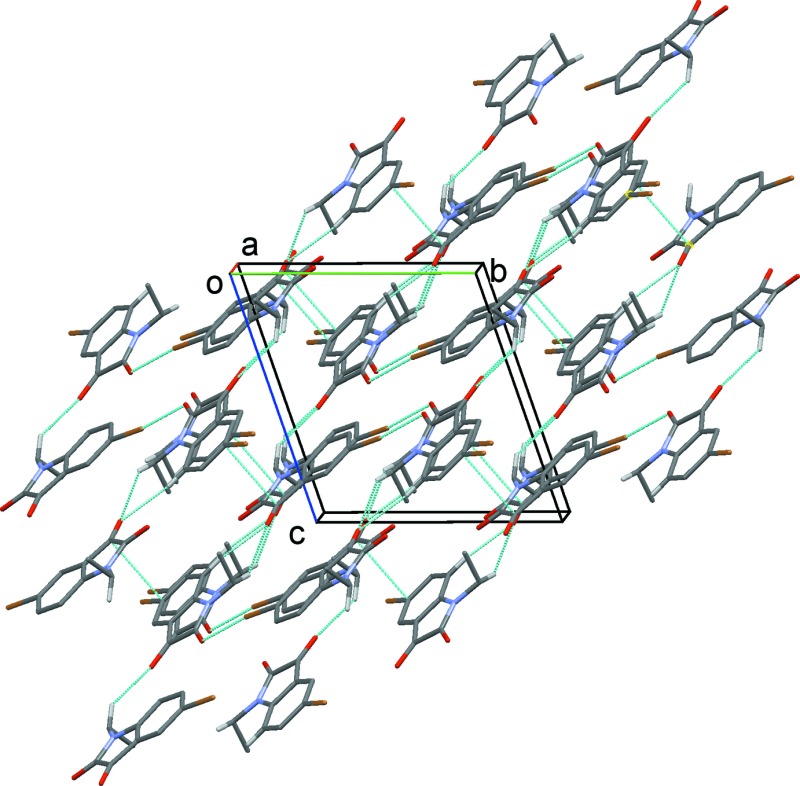
A view along the a axis of the crystal packing of the title compound. Hydrogen bonds (see Table 1) and other short interactions are shown as dashed lines. H atoms not involved in hydrogen bonding have been omitted for clarity.

. DOI: 10.1107/S2056989015023002/su5250fig1.tif
Mol­ecular structure of the title compound, with atom labelling. Displacement ellipsoids are drawn at the 50% probability level.

a . DOI: 10.1107/S2056989015023002/su5250fig2.tif
A view along the *a* axis of the crystal packing of the title compound. Hydrogen bonds (see Table 1) and other short inter­actions are shown as dashed lines. H atoms not involved in hydrogen bonding have been omitted for clarity.

CCDC reference: 1439717

Additional supporting information:  crystallographic information; 3D view; checkCIF report

## Figures and Tables

**Table 1:** Hydrogen-bond geometry (Å, °)

*D*—H⋯*A*	*D*—H	H⋯*A*	*D*⋯*A*	*D*—H⋯*A*
C9—H9*A*⋯O3^i^	0.97	2.58	3.351 (4)	136
C13—H13⋯O1^ii^	0.93	2.60	3.514 (3)	170
C19—H19*B*⋯O1^ii^	0.97	2.54	3.368 (3)	143

Symmetry codes: (i) 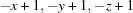; (ii) 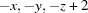.

## supplementary crystallographic information

### S1. Synthesis and crystallization

To 5-bromo-1*H*-indole-2,3-dione (0.4 g, 1.76 mmol) in DMF (25 ml) was added 1-bromo­ethane (0.21 ml, 1.93 mmol), potassium carbonate (0.6 g, 4.4 mmol), and a catalytic amount of tetra-n-butyl­ammonium bromide (0.1 g, 0.4 mmol). The mixture was stirred at room temperature for 48 h. The reaction was monitored by thin layer chromatography. On completion of the reaction the mixture was filtered and the solvent removed under vacuum. The title compound was obtained in 79% yield as red prismatic crystals (m.p. 409 K).

### S2. Structural commentary

5-bromo­isatin is an isatin derivative which has been reported to show a variety of biological activities, such as anti­bacterial, anti­microbial, anti­fungal and anti-HIV activities (Samus *et al.*, 2004; Sarangapani & Reddy, 1994; Varma *et al.*, 2004; Pandeya *et al.*, 1999). It has been used as a versatile reagent in organic synthesis, to obtain heterocyclic compounds, and as a raw material for drug synthesis (Abele *et al.*, 2003). Several isatin derivatives have been reported as being effective corrosion inhibitors for aluminium, copper and steel in different acid solution (Da Silva *et al.*, 2013).

The title compound, Fig. 1, crystallizes with two independent molecules (A and B) in the asymmetric unit. In each molecule the indoline ring system is almost planar with the largest deviation from the mean plane being 0.016 (2) Å for atom C8 in molecule A, and 0.040 (13) Å for atom C18 in molecule B. In each molecule, the ethyl group is nearly perpendicular to the indoline ring system as indicated by the torsion angles of C10–C9–N1–C8 = −94.8 (3)° and C20–C19–N2–C18 = −92.9 (3)°.

In the crystal, the two molecules are inclined to each other with a dihedral angle of 6.28 (8)°. The A and B molecules are linked to one another by C—H···O hydrogen bonds, forming –A—B—A—B– chains along direction [011]; see Table 1 and Fig. 2. Parallel chains are linked *via* a weak parallel slipped π–π inter­action [*Cg*1···*Cg*5^i^ = 3.6107 (14) Å, *Cg*1 and *Cg*5 are the centroids of rings (N1/C3/C4/C7/C8) and (C11—C16), respectively, inter-planar distance = 3.4584 (9) Å, slippage = 1.262 Å; symmetry code: (i) *x*, *y* − 1, *z*] and a short Br1···O4^ii^ contact [3.183 (2) Å; symmetry code: (ii) *x* − 1, *y*, *z*] forming a three-dimensional structure (Fig. 2).

### S3. Refinement

Crystal data, data collection and structure refinement details are summarized in Table 1. Crystal data, data collection and structure refinement details are summarized in Table 2. The H atoms were located in a difference Fourier map and treated as riding: C—H = 0.93–0.97 Å with *U*_iso_(H) = 1.5*U*_eq_(*C*-methyl) and 1.2*U*_eq_(C) for other H atoms. The reflection (1 0 0) affected by the beam stop was removed during the final cycles of refinement.

### Crystal data

**Table:**

C_10_H_8_BrNO_2_	*F*(000) = 504
*M**_r_* = 254.08	*D*_x_ = 1.714 Mg m^−^^3^
Triclinic, *P*1	Melting point: 409 K
*a* = 9.5198 (3) Å	Mo *K*α radiation, λ = 0.71073 Å
*b* = 10.0655 (3) Å	Cell parameters from 9899 reflections
*c* = 11.2341 (3) Å	θ = 2.4–26.9°
α = 70.9288 (16)°	µ = 4.15 mm^−^^1^
β = 75.4109 (16)°	*T* = 296 K
γ = 85.2199 (16)°	Prism, red
*V* = 984.58 (5) Å^3^	0.50 × 0.27 × 0.16 mm
*Z* = 4	

### Data collection

**Table:**

Bruker APEXII CCD diffractometer	4053 reflections with *I* > 2σ(*I*)
φ and ω scans	*R*_int_ = 0.045
Absorption correction: multi-scan (*SADABS*; Bruker, 2009)	θ_max_ = 31.1°, θ_min_ = 2.1°
*T*_min_ = 0.363, *T*_max_ = 0.746	*h* = −13→13
40192 measured reflections	*k* = −14→14
6275 independent reflections	*l* = −16→16

### Refinement

**Table:**

Refinement on *F*^2^	Primary atom site location: structure-invariant direct methods
Least-squares matrix: full	Secondary atom site location: difference Fourier map
*R*[*F*^2^ > 2σ(*F*^2^)] = 0.039	Hydrogen site location: inferred from neighbouring sites
*wR*(*F*^2^) = 0.105	H-atom parameters constrained
*S* = 1.02	*w* = 1/[σ^2^(*F*_o_^2^) + (0.0447*P*)^2^ + 0.4234*P*] where *P* = (*F*_o_^2^ + 2*F*_c_^2^)/3
6275 reflections	(Δ/σ)_max_ = 0.001
255 parameters	Δρ_max_ = 0.66 e Å^−^^3^
0 restraints	Δρ_min_ = −0.47 e Å^−^^3^

### Special details

**Table:**

Geometry. All e.s.d.'s (except the e.s.d. in the dihedral angle between two l.s. planes) are estimated using the full covariance matrix. The cell e.s.d.'s are taken into account individually in the estimation of e.s.d.'s in distances, angles and torsion angles; correlations between e.s.d.'s in cell parameters are only used when they are defined by crystal symmetry. An approximate (isotropic) treatment of cell e.s.d.'s is used for estimating e.s.d.'s involving l.s. planes.

### Fractional atomic coordinates and isotropic or equivalent isotropic displacement parameters (Å^2^)

**Table:**

	*x*	*y*	*z*	*U*_iso_*/*U*_eq_	
Br1	−0.18497 (3)	0.38373 (3)	0.65426 (3)	0.05961 (10)	
Br2	−0.21774 (3)	0.83177 (4)	0.67449 (3)	0.06693 (11)	
C1	−0.0541 (3)	0.2362 (3)	0.7041 (2)	0.0455 (5)	
C2	−0.1069 (2)	0.1111 (3)	0.7981 (2)	0.0448 (5)	
H2	−0.2058	0.0980	0.8355	0.054*	
C3	−0.0083 (2)	0.0065 (2)	0.8345 (2)	0.0401 (5)	
C4	0.1408 (2)	0.0245 (2)	0.7783 (2)	0.0398 (5)	
C5	0.1932 (3)	0.1482 (3)	0.6833 (2)	0.0470 (5)	
H5	0.2918	0.1603	0.6445	0.056*	
C6	0.0941 (3)	0.2541 (3)	0.6475 (2)	0.0490 (6)	
H6	0.1273	0.3387	0.5844	0.059*	
C7	−0.0281 (3)	−0.1350 (2)	0.9294 (2)	0.0452 (5)	
C8	0.1282 (3)	−0.1971 (2)	0.9241 (2)	0.0457 (5)	
C9	0.3772 (3)	−0.1079 (3)	0.8006 (3)	0.0492 (6)	
H9A	0.4152	−0.0651	0.7081	0.059*	
H9B	0.4041	−0.2065	0.8230	0.059*	
C10	0.4440 (3)	−0.0384 (4)	0.8721 (3)	0.0638 (7)	
H10A	0.4160	0.0588	0.8516	0.096*	
H10B	0.5478	−0.0453	0.8469	0.096*	
H10C	0.4112	−0.0842	0.9637	0.096*	
C11	−0.0389 (2)	0.7381 (3)	0.6866 (2)	0.0456 (5)	
C12	−0.0362 (3)	0.6079 (3)	0.7812 (2)	0.0476 (5)	
H12	−0.1223	0.5695	0.8391	0.057*	
C13	0.0932 (3)	0.5346 (2)	0.7905 (2)	0.0446 (5)	
H13	0.0952	0.4482	0.8540	0.054*	
C14	0.2183 (2)	0.5947 (2)	0.7023 (2)	0.0384 (5)	
C15	0.2160 (2)	0.7266 (2)	0.6083 (2)	0.0420 (5)	
C16	0.0875 (3)	0.7996 (3)	0.5993 (2)	0.0483 (5)	
H16	0.0858	0.8870	0.5369	0.058*	
C17	0.3641 (3)	0.7565 (3)	0.5299 (2)	0.0503 (6)	
C18	0.4542 (3)	0.6263 (3)	0.5872 (2)	0.0471 (5)	
C19	0.4003 (3)	0.4048 (3)	0.7749 (3)	0.0532 (6)	
H19A	0.4842	0.3664	0.7274	0.064*	
H19B	0.3210	0.3386	0.8024	0.064*	
C20	0.4349 (4)	0.4217 (4)	0.8918 (3)	0.0863 (11)	
H20A	0.5182	0.4814	0.8652	0.129*	
H20B	0.4552	0.3314	0.9481	0.129*	
H20C	0.3535	0.4634	0.9371	0.129*	
N1	0.2194 (2)	−0.09608 (19)	0.83095 (18)	0.0435 (4)	
N2	0.3599 (2)	0.5384 (2)	0.68952 (18)	0.0436 (4)	
O1	−0.1362 (2)	−0.1955 (2)	1.00032 (18)	0.0615 (5)	
O2	0.1621 (2)	−0.31109 (18)	0.98927 (18)	0.0608 (5)	
O3	0.4154 (2)	0.8572 (2)	0.4392 (2)	0.0769 (7)	
O4	0.58302 (19)	0.6081 (2)	0.54841 (19)	0.0622 (5)	

### Atomic displacement parameters (Å^2^)

**Table:**

	*U*^11^	*U*^22^	*U*^33^	*U*^12^	*U*^13^	*U*^23^
Br1	0.05388 (16)	0.05547 (16)	0.06915 (19)	0.00842 (12)	−0.01909 (13)	−0.01804 (13)
Br2	0.04361 (15)	0.0816 (2)	0.0751 (2)	0.01261 (14)	−0.01524 (13)	−0.02645 (16)
C1	0.0427 (12)	0.0441 (12)	0.0496 (13)	0.0017 (10)	−0.0109 (10)	−0.0153 (10)
C2	0.0367 (11)	0.0503 (13)	0.0456 (12)	−0.0072 (10)	−0.0014 (9)	−0.0176 (10)
C3	0.0395 (11)	0.0406 (11)	0.0368 (11)	−0.0092 (9)	−0.0006 (9)	−0.0119 (9)
C4	0.0405 (11)	0.0405 (11)	0.0362 (11)	−0.0058 (9)	−0.0034 (9)	−0.0121 (9)
C5	0.0384 (12)	0.0483 (13)	0.0440 (12)	−0.0095 (10)	0.0014 (9)	−0.0071 (10)
C6	0.0454 (13)	0.0440 (12)	0.0466 (13)	−0.0079 (10)	−0.0037 (10)	−0.0032 (10)
C7	0.0473 (13)	0.0430 (12)	0.0412 (12)	−0.0126 (10)	0.0001 (10)	−0.0128 (10)
C8	0.0533 (14)	0.0414 (12)	0.0395 (12)	−0.0087 (10)	−0.0026 (10)	−0.0130 (10)
C9	0.0402 (12)	0.0479 (13)	0.0533 (14)	0.0035 (10)	−0.0020 (10)	−0.0156 (11)
C10	0.0444 (14)	0.079 (2)	0.0712 (18)	0.0016 (13)	−0.0120 (13)	−0.0304 (16)
C11	0.0372 (11)	0.0518 (13)	0.0497 (13)	0.0045 (10)	−0.0077 (10)	−0.0218 (11)
C12	0.0398 (12)	0.0504 (13)	0.0462 (12)	−0.0078 (10)	0.0025 (10)	−0.0148 (11)
C13	0.0434 (12)	0.0400 (11)	0.0416 (12)	−0.0066 (10)	0.0000 (9)	−0.0073 (9)
C14	0.0386 (11)	0.0366 (10)	0.0368 (11)	−0.0045 (9)	−0.0031 (9)	−0.0106 (9)
C15	0.0398 (11)	0.0403 (11)	0.0388 (11)	−0.0054 (9)	−0.0022 (9)	−0.0073 (9)
C16	0.0463 (13)	0.0442 (12)	0.0470 (13)	0.0003 (10)	−0.0094 (10)	−0.0059 (10)
C17	0.0436 (13)	0.0478 (13)	0.0470 (13)	−0.0088 (11)	−0.0011 (10)	−0.0037 (11)
C18	0.0405 (12)	0.0477 (13)	0.0470 (12)	−0.0072 (10)	−0.0013 (10)	−0.0119 (10)
C19	0.0472 (14)	0.0402 (12)	0.0577 (15)	0.0041 (11)	−0.0044 (11)	−0.0036 (11)
C20	0.094 (3)	0.093 (3)	0.0634 (19)	0.022 (2)	−0.0306 (18)	−0.0099 (18)
N1	0.0414 (10)	0.0396 (10)	0.0433 (10)	−0.0044 (8)	−0.0031 (8)	−0.0090 (8)
N2	0.0373 (10)	0.0402 (10)	0.0427 (10)	−0.0018 (8)	−0.0001 (8)	−0.0059 (8)
O1	0.0537 (11)	0.0562 (11)	0.0576 (11)	−0.0199 (9)	0.0069 (8)	−0.0059 (9)
O2	0.0708 (13)	0.0416 (9)	0.0563 (11)	−0.0040 (9)	−0.0075 (9)	−0.0022 (8)
O3	0.0518 (11)	0.0630 (12)	0.0751 (14)	−0.0094 (10)	0.0033 (10)	0.0209 (10)
O4	0.0382 (9)	0.0634 (12)	0.0676 (12)	−0.0040 (9)	0.0016 (8)	−0.0073 (10)

### Geometric parameters (Å, º)

**Table:**

Br1—C1	1.889 (2)	C10—H10C	0.9600
Br2—C11	1.890 (2)	C11—C16	1.388 (3)
C1—C2	1.387 (3)	C11—C12	1.395 (4)
C1—C6	1.394 (3)	C12—C13	1.391 (4)
C2—C3	1.379 (3)	C12—H12	0.9300
C2—H2	0.9300	C13—C14	1.378 (3)
C3—C4	1.401 (3)	C13—H13	0.9300
C3—C7	1.467 (3)	C14—C15	1.403 (3)
C4—C5	1.381 (3)	C14—N2	1.410 (3)
C4—N1	1.409 (3)	C15—C16	1.382 (3)
C5—C6	1.390 (4)	C15—C17	1.458 (3)
C5—H5	0.9300	C16—H16	0.9300
C6—H6	0.9300	C17—O3	1.209 (3)
C7—O1	1.200 (3)	C17—C18	1.551 (4)
C7—C8	1.560 (4)	C18—O4	1.214 (3)
C8—O2	1.210 (3)	C18—N2	1.363 (3)
C8—N1	1.369 (3)	C19—N2	1.461 (3)
C9—N1	1.458 (3)	C19—C20	1.495 (4)
C9—C10	1.497 (4)	C19—H19A	0.9700
C9—H9A	0.9700	C19—H19B	0.9700
C9—H9B	0.9700	C20—H20A	0.9600
C10—H10A	0.9600	C20—H20B	0.9600
C10—H10B	0.9600	C20—H20C	0.9600
			
C2—C1—C6	120.6 (2)	C13—C12—C11	121.1 (2)
C2—C1—Br1	119.35 (18)	C13—C12—H12	119.4
C6—C1—Br1	120.06 (18)	C11—C12—H12	119.4
C3—C2—C1	118.0 (2)	C14—C13—C12	117.6 (2)
C3—C2—H2	121.0	C14—C13—H13	121.2
C1—C2—H2	121.0	C12—C13—H13	121.2
C2—C3—C4	121.6 (2)	C13—C14—C15	121.3 (2)
C2—C3—C7	131.4 (2)	C13—C14—N2	127.9 (2)
C4—C3—C7	107.0 (2)	C15—C14—N2	110.87 (18)
C5—C4—C3	120.5 (2)	C16—C15—C14	121.1 (2)
C5—C4—N1	128.3 (2)	C16—C15—C17	131.9 (2)
C3—C4—N1	111.24 (19)	C14—C15—C17	107.0 (2)
C4—C5—C6	118.0 (2)	C15—C16—C11	117.7 (2)
C4—C5—H5	121.0	C15—C16—H16	121.2
C6—C5—H5	121.0	C11—C16—H16	121.2
C5—C6—C1	121.4 (2)	O3—C17—C15	131.2 (3)
C5—C6—H6	119.3	O3—C17—C18	123.6 (2)
C1—C6—H6	119.3	C15—C17—C18	105.25 (19)
O1—C7—C3	130.6 (2)	O4—C18—N2	127.4 (2)
O1—C7—C8	124.4 (2)	O4—C18—C17	126.4 (2)
C3—C7—C8	105.00 (18)	N2—C18—C17	106.18 (19)
O2—C8—N1	127.0 (2)	N2—C19—C20	111.7 (2)
O2—C8—C7	127.0 (2)	N2—C19—H19A	109.3
N1—C8—C7	106.0 (2)	C20—C19—H19A	109.3
N1—C9—C10	112.1 (2)	N2—C19—H19B	109.3
N1—C9—H9A	109.2	C20—C19—H19B	109.3
C10—C9—H9A	109.2	H19A—C19—H19B	107.9
N1—C9—H9B	109.2	C19—C20—H20A	109.5
C10—C9—H9B	109.2	C19—C20—H20B	109.5
H9A—C9—H9B	107.9	H20A—C20—H20B	109.5
C9—C10—H10A	109.5	C19—C20—H20C	109.5
C9—C10—H10B	109.5	H20A—C20—H20C	109.5
H10A—C10—H10B	109.5	H20B—C20—H20C	109.5
C9—C10—H10C	109.5	C8—N1—C4	110.77 (19)
H10A—C10—H10C	109.5	C8—N1—C9	123.9 (2)
H10B—C10—H10C	109.5	C4—N1—C9	125.04 (18)
C16—C11—C12	121.2 (2)	C18—N2—C14	110.69 (19)
C16—C11—Br2	119.07 (19)	C18—N2—C19	124.7 (2)
C12—C11—Br2	119.72 (18)	C14—N2—C19	124.64 (18)
			
C6—C1—C2—C3	0.8 (4)	C17—C15—C16—C11	176.5 (3)
Br1—C1—C2—C3	−178.63 (17)	C12—C11—C16—C15	0.8 (4)
C1—C2—C3—C4	−0.4 (3)	Br2—C11—C16—C15	−178.19 (18)
C1—C2—C3—C7	179.7 (2)	C16—C15—C17—O3	4.3 (5)
C2—C3—C4—C5	−0.6 (3)	C14—C15—C17—O3	−179.0 (3)
C7—C3—C4—C5	179.3 (2)	C16—C15—C17—C18	−176.1 (3)
C2—C3—C4—N1	179.9 (2)	C14—C15—C17—C18	0.6 (3)
C7—C3—C4—N1	−0.2 (3)	O3—C17—C18—O4	−1.4 (5)
C3—C4—C5—C6	1.1 (3)	C15—C17—C18—O4	179.0 (3)
N1—C4—C5—C6	−179.4 (2)	O3—C17—C18—N2	178.1 (3)
C4—C5—C6—C1	−0.8 (4)	C15—C17—C18—N2	−1.6 (3)
C2—C1—C6—C5	−0.2 (4)	O2—C8—N1—C4	−178.3 (2)
Br1—C1—C6—C5	179.2 (2)	C7—C8—N1—C4	1.4 (2)
C2—C3—C7—O1	−0.3 (4)	O2—C8—N1—C9	−3.8 (4)
C4—C3—C7—O1	179.8 (3)	C7—C8—N1—C9	175.9 (2)
C2—C3—C7—C8	−179.1 (2)	C5—C4—N1—C8	179.7 (2)
C4—C3—C7—C8	1.1 (2)	C3—C4—N1—C8	−0.8 (3)
O1—C7—C8—O2	−0.6 (4)	C5—C4—N1—C9	5.2 (4)
C3—C7—C8—O2	178.2 (2)	C3—C4—N1—C9	−175.2 (2)
O1—C7—C8—N1	179.6 (2)	C10—C9—N1—C8	−94.8 (3)
C3—C7—C8—N1	−1.5 (2)	C10—C9—N1—C4	78.8 (3)
C16—C11—C12—C13	−0.7 (4)	O4—C18—N2—C14	−178.6 (3)
Br2—C11—C12—C13	178.34 (19)	C17—C18—N2—C14	2.0 (3)
C11—C12—C13—C14	−0.5 (4)	O4—C18—N2—C19	1.8 (4)
C12—C13—C14—C15	1.5 (3)	C17—C18—N2—C19	−177.6 (2)
C12—C13—C14—N2	−177.4 (2)	C13—C14—N2—C18	177.3 (2)
C13—C14—C15—C16	−1.4 (4)	C15—C14—N2—C18	−1.7 (3)
N2—C14—C15—C16	177.7 (2)	C13—C14—N2—C19	−3.1 (4)
C13—C14—C15—C17	−178.5 (2)	C15—C14—N2—C19	177.9 (2)
N2—C14—C15—C17	0.6 (3)	C20—C19—N2—C18	93.0 (3)
C14—C15—C16—C11	0.2 (4)	C20—C19—N2—C14	−86.5 (3)

### Hydrogen-bond geometry (Å, º)

**Table:**

*D*—H···*A*	*D*—H	H···*A*	*D*···*A*	*D*—H···*A*
C9—H9*A*···O3^i^	0.97	2.58	3.351 (4)	136
C13—H13···O1^ii^	0.93	2.60	3.514 (3)	170
C19—H19*B*···O1^ii^	0.97	2.54	3.368 (3)	143

Symmetry codes: (i) −*x*+1, −*y*+1, −*z*+1; (ii) −*x*, −*y*, −*z*+2.
