# Supplementary figures and images for: Crystal structure of 5-bromo-1-ethyl­indoline-2,3-dione
Source: Acta Crystallogr E Crystallogr Commun. 2015 Dec 6;71(Pt 12):o1024–5. doi: 10.1107/S2056989015023002 (PMC4719959; doi:10.1107/S2056989015023002)

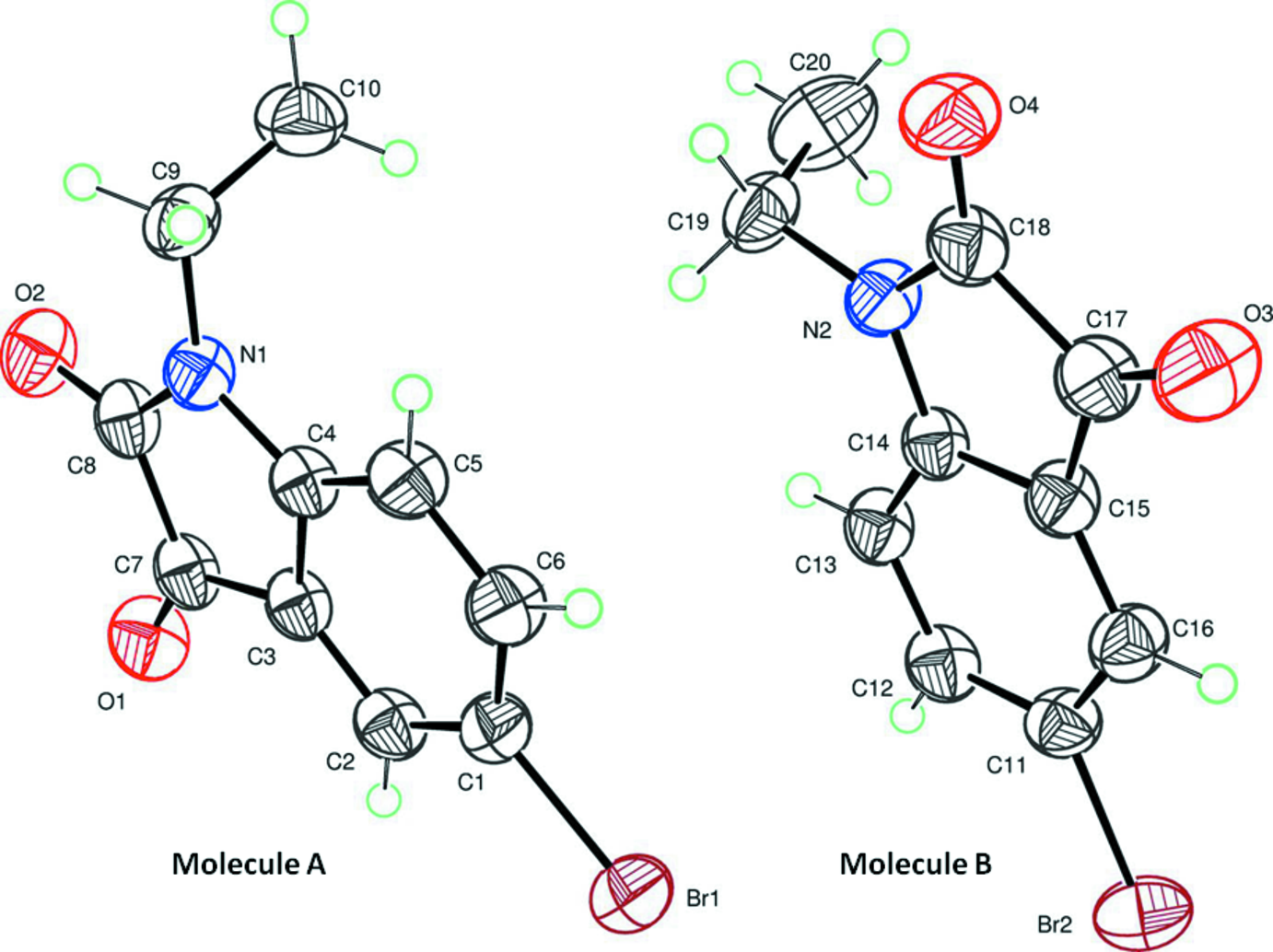

Supplement: Supplementary file 4 [file e-71-o1024-fig1.tif]

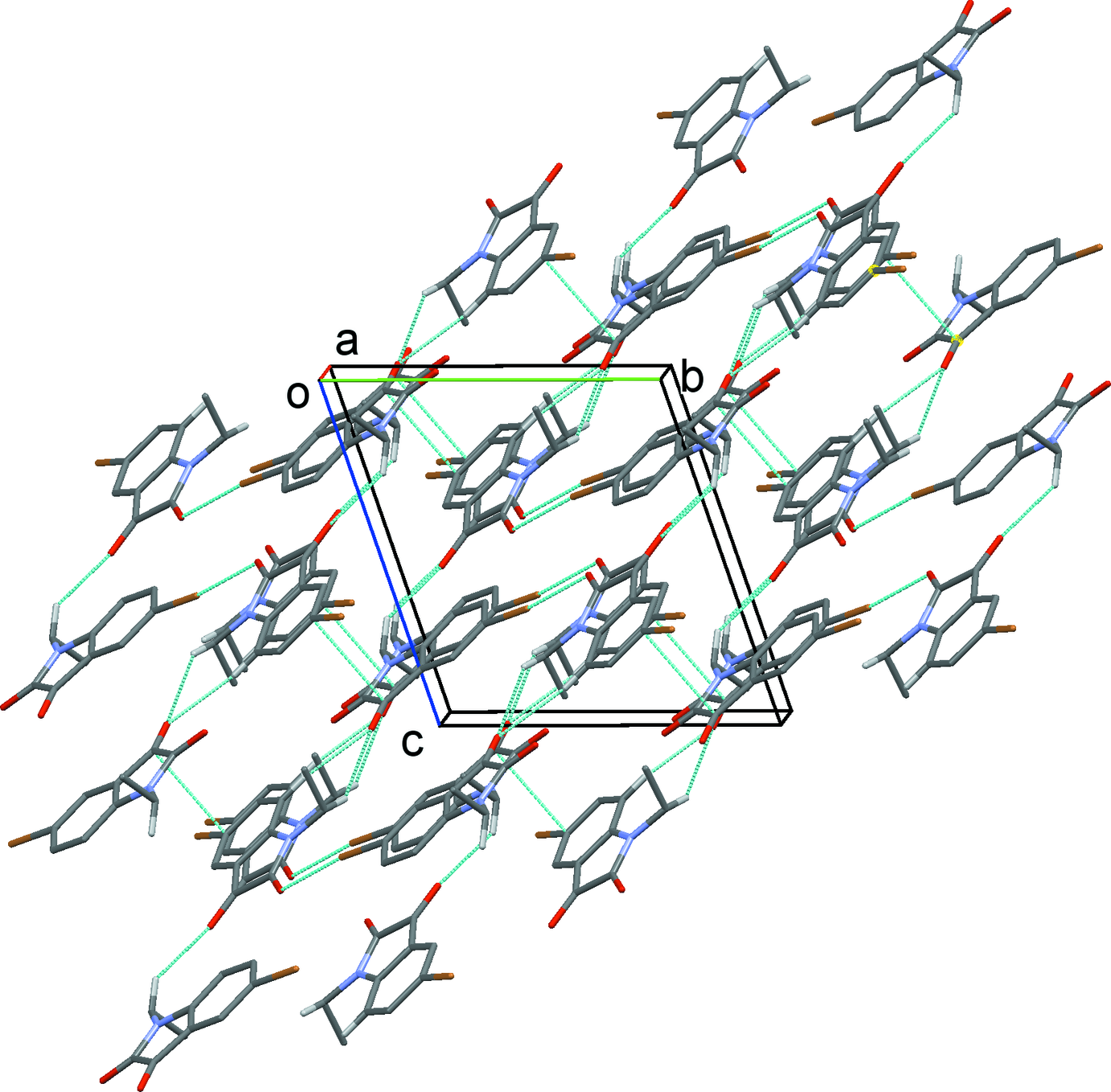

Supplement: Supplementary file 5 [file e-71-o1024-fig2.tif]
